# Thermo-responsive poly(*N*-isopropylacrylamide)-*block*-poly(ionic liquid) of pyridinium sulfonate immobilized Pd nanoparticles in C–C coupling reactions†

**DOI:** 10.1039/c8ra01303a

**Published:** 2018-04-18

**Authors:** Soheila Ghasemi, Zahra Amini Harandi

**Affiliations:** Department of Chemistry, College of Sciences, Shiraz University Shiraz Iran ghasemis@shirazu.ac.ir

## Abstract

A thermo-responsive poly(*N*-isopropylacrylamide)-*block*-poly(ionic liquid) (PNIPAM-*b*-PIL) of pyridinium-type was prepared. Initially, controlled synthesis of PNIPAM was performed *via* RAFT method. Subsequently, PNIPAM as macromolecular chain transfer agent (macro-CTA) was used for fabrication of PNIPAM-*b*-PIL through reaction with a synthesized IL monomer *i.e.* 4-vinyl pyridinium propane sulfonate. The Pd catalyst was produced throughout palladium nanoparticles' anchoring into this block copolymer. The catalyst was characterized using ICP, FT-IR, NMR, UV-Vis, TGA, XRD, SEM and EDX techniques. The catalyst's TEM image proved nearly fine dispersion of PdNPs with negligible agglomeration. The catalyst was used in the production of a variety of substituted alkenes and biaryl compounds (Heck and Suzuki coupling) in organic and aqueous media and under solvent free conditions. Additionally, the results signified extreme reusability of the catalyst with a simple recycling procedure.

## Abstract 2

Preparation of thermo-responsive PNIPAM-*b*-PIL/PdNPs *via* RAFT method and its catalytic behavior in C–C coupling with extreme reusability.

## Graphical abstract

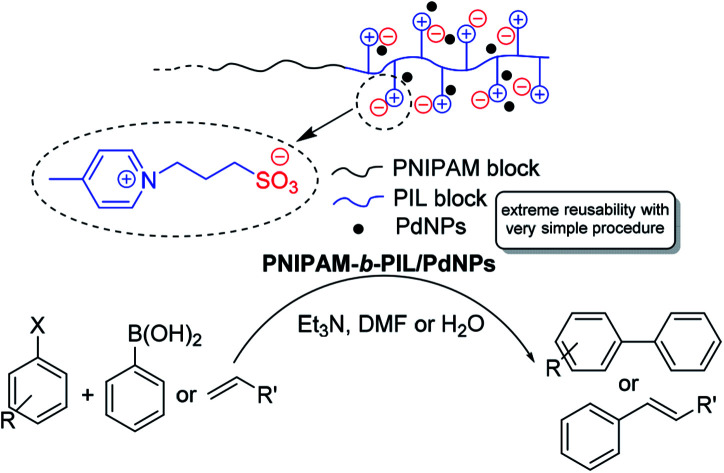

## 1. Introduction

Block copolymers are an appealing type of macromolecule with significant profit in technological applications and theoretical exploration.^1^ Outstanding electrical, mechanical, magnetic, and acoustic properties of block copolymers are the result of their micro phase separation and self-assembly into 3D highly ordered nanostructures. Among diverse polymerization systems *i.e.* free radical, anionic, cationic, coordination, group transfer, *etc* for the synthesis of block copolymers, controlled radical polymerization (CRP) is the most extensively used approach.^2,3^ The most applied CRP strategies for the production of well-defined (co)polymers are atom transfer radical polymerization (ATRP),^4,5^ nitroxide-mediated polymerization (NMP),^6^ and reversible addition–fragmentation chain transfer (RAFT) polymerization.^7–10^ However, employing NMP is restricted to the monomer type and condition of polymerization and a bit of metal catalyst persisting in the ultimate product has limited the utilization of ATRP in biomedical and electronic applications. In this regard, RAFT has demonstrated as exceedingly successful technique for the synthesis of block copolymers.^11^ For instance, block copolymers of 1,2,4-triazolium-based polymers with remarkable ion conductivity,^12^ lipid-based hydrophilic-hydrophobic block copolymer as nano drug carrier^13^ and thermo-responsive star block copolymers of *N*,*N*-dimethyl and diethyl acrylamide (DMA and DEA)^14^ were synthesized through RAFT polymerization.

Monomers of ionic liquid (IL) produces a remarkable kind of polyelectrolytes, which are entitled poly(ionic liquid)s (PILs) with enormous potential applications.^15–18^ PIL emerged the property of IL and polymer in order to generate novel characters and functions. The significant benefits of PILs compare to ILs are enhanced mechanical stability, processability and strength and structural control over the IL groups.

Due to the extraordinary characteristics of block copolymers and PILs, the preparation of block copolymers include at least single PIL segment has merit of remarkable notice.^19^ However, by means of choosing one thermo-responsive block, it is possible to study thermal phase behavior of these systems.^20,21^ Generally, PIL block copolymers were synthesized through CRP techniques such as RAFT and ATRP.^22,23^ However, the reactivity of IL monomer ought to be adapted with these polymerization techniques. Texter *et al.* was announced the use of ATRP for the production of ABA triblock terpolymer with symmetric side chains PILs blocks of imidazolium type using bifunctional poly(propylene oxide) by macro ATRP initiator termini.^24^ Triblock copolymers with short cationic PIL end blocks and a PNIPAM middle block were prepared using ATRP.^25^ Gu and Lodge was announced the utilization of RAFT for the preparation of ABA triblock terpolymer with the middle imidazolium based PIL block.^26^ Thermo responsive diblock copolymers consist of a PNIPAM and PIL segments have been synthesized *via* RAFT method.^27^ Moreover, NMP was employed appropriately for the production of imidazolium and phosphonium class of PIL diblock copolymers or triblock terpolymers.^28,29^

PILs can complex and stabilize catalytically active metal nanoparticles strongly and perpetually due to the excessive charge density and polymer architecture.^30–32^ In addition, PIL-based systems assisted the recovery and further use of the supports.

Pd-catalyzed carbon–carbon cross coupling as Heck and Suzuki in ILs was achieved remarkable progress in the past decade.^33–35^ A great number of Pd coupling assisted in ILs *i.e.* tetraalkyl-ammonium, phosphonium, imidazolium and pyridinium-based systems can be found in the following reviews.^36–39^ In addition, supported ILs are developed in order to provide robust, reusable and efficient catalysts.^40,41^ Selected examples are; 1-aminoethyl-3-vinylimidazolium bromide ([VAIM]Br) attached on the macromolecular network supported PdNPs^42^ and Pd immobilized on poly(1-aminoethyl-3-vinylimidazolium bromide) coupled with magnetic nanoparticles (Pd/Fe_3_O_4_@PIL-NH_2_) for solvent-free Heck reaction,^43^ palladium catalysts anchored on gel-supported ionic liquid-like phases (g-SILLPs) in Heck coupling,^44^ assembly of PdCl_2_ in IL brushes for coupling of iodoarenes with acrylic acid in water,^45^ IL/Pd(OAc)_2_ on the mesoporous SBA-15 for Heck reaction,^46^*N*-heterocyclic carbene (NHC)/Pd supported on IL-functionalized graphene oxide for Suzuki coupling,^47^ NHC/Pd complex supported IL-modified SBA-16 for the Suzuki and Heck reactions,^48^ functional IL modified Fe_3_O_4_/PdNPs for biaryl formation *via* Suzuki coupling^49^ and PIL entrapped magnetic nanoparticles/Pd for the solvent-free Heck reaction.^50^

In continuation of our previous report on the preparation of different type of heterogeneous Pd catalysts,^51–53^ and considering the outstanding characteristics of block copolymers and PILs, herein we reported a proper strategy for the preparation of thermo-responsive PNIPAM-*b*-PIL *via* RAFT method. The corresponding Pd catalyst was produced through the immobilization of PdNPs onto this block copolymer. Afterwards, the potency of the Pd catalyst was examined in C–C coupling (Mizoroki–Heck and Suzuki–Miyaura).

## 2. Experimental

### 2.1. General remarks

#### 2.1.1. Materials

Azobisisobutyronitrile (AIBN, Merck, 98%) and *N*-isopropylacrylamide (NIPAM, Acros, 99%) purification was performed *via* re-crystallization from methanol and *n*-hexane respectively. 4-Vinylpyridine (4-VP, Sigma-Aldrich, 95%) was refined through vacuum distillation. 2-(Dodecylthiocarbonothioylthio)-2-methylpropionic acid (DDMAT, Sigma-Aldrich, 98% HPLC) make dried under vacuum, preliminary to use. 1,4-Dioxane (Fluka, 99.5%) dried initially using CaCl_2_ and then by refluxing in the presence of sodium wire. 1,3-Propanesultone (Sigma-Aldrich, 98%) was used as obtained.

#### 2.1.2. Characterization systems

Coupling products were recognized by FT-IR and NMR and comparison with genuine specimens. Monitoring the progress of the coupling reactions performed by thin layer chromatography (TLC) using silica-gel plate (polygram SIL/UV 254). UV-Visible (CECIL CE7250 spectrophotometer), IR (Shimadzu FTIR-8300 instrument) and NMR (250 or 400 MHz Bruker Avance DPX apparatus in acetone-*d*_6_ or D_2_O) spectroscopy were used for collecting the corresponding spectra. Varian, Vista-Pro ICP-OES analyzer was employed for measuring the Pd content of the catalyst. TG/DTA data (Perkin-Elmer Pyris Diamond appliance) was derived under the condition of r.t up to 800 °C temperature range and heating ramp rate of 10 °C min^−1^ under N_2_ gas in Pt pans. X-Ray powder diffraction (XRD) graphs was gained using X-ray diffractometer (Bruker AXS D8-Advance, Cu Kα radiation of *λ* = 1.541874 Å and 10 to 90° of 2*θ* scan range at ambient temperature). Determining polymer ingredient was achieved by energy-dispersive X-ray spectroscopy (EDX, VEGA3 TESCAN). Scanning electron images were gathered on VEGA3 TESCAN microscope at 20 kV. Gold coating before SEM photography was implemented by VEGA3 TESCAN sputter coater. Transmission electron pictures acquired by 200 kV JEOL, JEM-2100F Philips TEM implement. Elemental analyzer of FIASHEA 1112 thermo finningan model was used for investigation of elemental composition of specimens. Hydrodynamic size of polymers and the charge of ionic liquids were achieved through dynamic light scattering (DLS) (NANO-flex) and zeta seizer (ZETA-check Particle Charge Reader) respectively.

### 2.2. Poly(*N*-isopropylacrylamide-*b*-ionic liquid) (PNIPAM-*b*-PIL) supported PdNPs

#### 2.2.1. PNIPAM preparation *via* RAFT procedure

In a Schelenk reaction tube, monomer (NIPAM, 100.00 mmol, 11.31 g), chain transfer agent (DDMAT, 1.00 mmol, 0.3646 g), initiator (AIBN, 0.200 mmol, 0.0328 g) and 20 mL of dry 1,4-dioxane were mixed. The reaction tube was purged with Ar for 15–30 min and then degassed for 5 to 7 times through freeze–evacuate–thaw cycle. The Schelenk tube was sealed and heated at 80 °C for 12 h. The PNIPAM-CTA was separated by precipitation in dry *n*-hexane twice, after re-dissolution in dioxane. The resultant product was desiccated in vacuum at 60 °C before being used for the next step.

#### 2.2.2. 4-Vinyl pyridinium propane sulfonate preparation

For the synthesis of monomer of ionic liquid of 4-vinylpyridine (4-VP), a 50 mL round bottom flask at an ice bath was charged with 4-vinylpyridine (100 mmol, 10.87 mL) in 20 mL of methanol. Afterward, 1,3-propanesultone (100 mmol, 8.77 mL) was added in small quantity during 10 minutes. The corresponding amalgam was stirred for additional 5 h at ambient temperature. The yellow product was collected and washed thoroughly with dry diethyl ether and then desiccated under vacuum overnight.

#### 2.2.3. Thermo-responsive PNIPAM-*b*-PIL

The polymerization of ionic liquid was originally performed using the thiocarbonothioylthio-terminated PNIPAM as macro-CTA. The PNIPAM-CTA (0.03 mmol, 344 mg), AIBN (0.2 mmol, 0.98 mg) and 4-vinyl pyridinium propane sulfonate (3 mmol, 682 mg) in 3 mL dry 1,4-dioxane were placed in a Schelenk reaction tube. All solvent degassing procedure, heating the mixture, product precipitation and drying was repeated as expressed in Section 2.2.1 to isolate the PNIPAM-*b*-PIL.

#### 2.2.4. Thermo-responsive PNIPAM-*b*-PIL/Pd catalyst

The PNIPAM-*b*-PIL (0.16 mmol, 350 mg) in DMF (3 mL) and Pd(OAc)_2_ (0.44 mmol, 99 mg) was added into a 10 mL round-bottom flask and stirred for 24 h at room temperature. The corresponding mixture was next precipitated in dry diethyl ether twice. The brown product was gathered and desiccated in vacuum overnight. Black Pd (0) nanoparticles supported on block copolymer were yielded by adding NaBH_4_ (0.264 mmol, 10 mg) as a reducing agent into a PNIPAM-*b*-PIL in CH_3_OH (10 mL) and agitated for 24 h at ambient temperature. The suspension was next precipitated in dry diethyl ether and the black solid was accumulated and desiccated in vacuum overnight.

### 2.3. Cross-coupling reactions

#### 2.3.1. Mizoroki–Heck coupling; general procedure in H_2_O and DMF

A test tube equipped with a magnet was loaded with haloarene (1.0 mmol), *n*-butyl acrylate or styrene (1.2 mmol), Et_3_N (2.0 mmol, 0.279 mL) and Pd catalyst (0.1 mol%, 7.8 mg) in H_2_O (0.5 mL) or DMF (2 mL). The flask was located in an oil bath and stirred and heated at 85 °C for an appropriate time. The reaction's progress was monitored by TLC. After ending of the reaction, the suspension was chilled to ambient temperature and dry diethyl ether was added to the amalgam. In water as solvent, the product extracted to organic phase and the catalyst remained dissolved in water. The aqueous phase was washed with diethyl ether three times and the combined organic phases evaporated after drying over anhydrous MgSO_4_. In DMF as solvent, the catalyst was precipitated after diethyl ether addition and recovered by filtration. The organic phase was washed with H_2_O and next evaporated and dried. The compound was then refined by plate chromatography over silica gel in petroleum ether (90) : ethyl acetate (10) as eluent to afford the coupling product. The aryl-alkene products characterization was carried out by comparing their spectral data with the authentic specimens.

#### 2.3.2. Suzuki–Miyaura coupling; general procedure in H_2_O

In a test tube, phenylboronic acid (1.2 mmol, 0.146 g) was added to the suspension include haloarene (1.0 mmol), Et_3_N (2.0 mmol, 0.279 mL) and Pd catalyst (0.1 mol%, 7.8 mg) in H_2_O (0.5 mL). The mixture was agitated and heated at 85 °C for a proper time. Development of the reactions was checked using TLC until disappearing of starting haloarene. After reaction completion, the above mentioned procedure (Section 2.3.1) was repeated for recovery and characterization of biaryl compounds.

#### 2.3.3. Catalyst recycling after a coupling reaction

##### 2.3.3.1. In organic media (DMF)

After a Heck reaction in DMF, the suspension was chilled to ambient temperature and diethyl ether was added to the mixture. The product was remained in organic phase and the catalyst was precipitated. After solvent decanting, the recovered catalyst was reused in the subsequent reaction run with a pristine amount of substrates with no pretreatment. The Pd catalyst was used more than 20 cycles and maintained its catalytic function in the repeating sequences.

##### 2.3.3.2. In aqueous media

Following a Heck or Suzuki reaction in water, the reaction mixture was chilled to ambient temperature and diethyl ether was added to the suspension. The product extracted to organic phase and the catalyst remained in water. Two phases were isolated and the aqueous phase was washed with diethyl ether three times. Afterward, to a mixture of recycled Pd catalyst in H_2_O was added new quantity of reactants. The Pd catalyst was used for 10 cycles in coupling reactions and preserved its activity in the repeating runs.

## 3. Result and discussion

### 3.1. Thermo-responsive PNIPAM-*b*-PIL containing PdNPs *via* RAFT polymerization; preparation and characterization

**Scheme 1:**
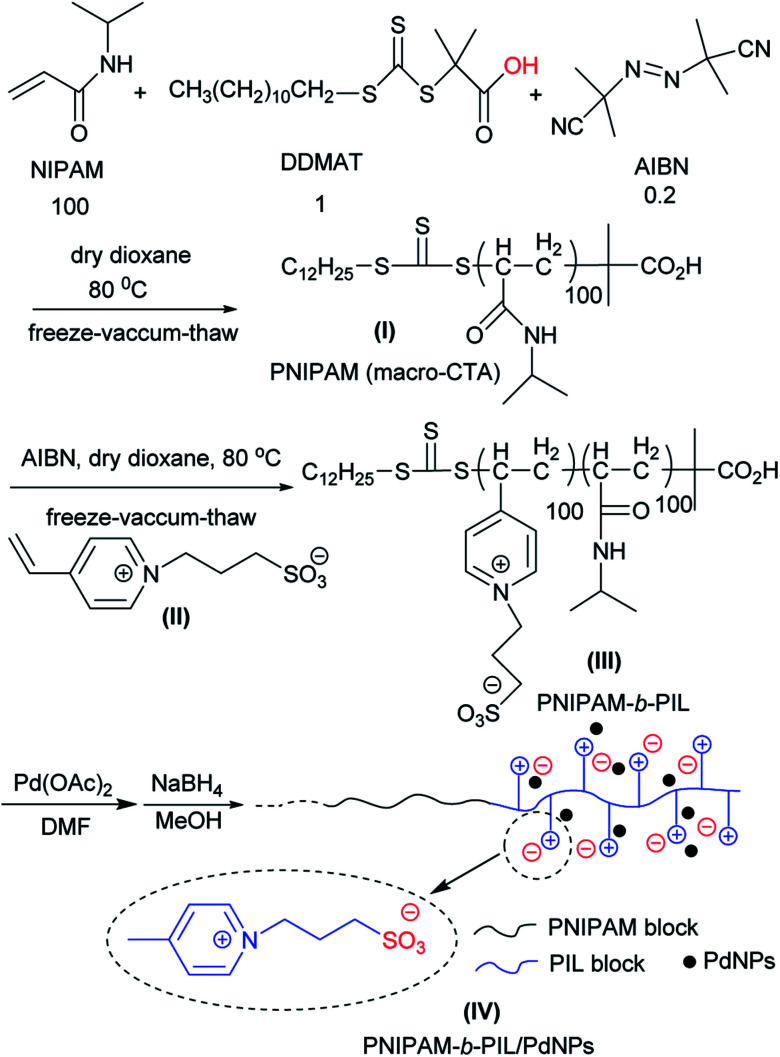
Schematic representation for the preparation of thermo-responsive PNIPAM-*b*-PIL/PdNPs.

PNIPAM-*b*-PIL as a thermo-responsive block polymeric support was prepared through RAFT polymerization system (Scheme 1). Originally, NIPAM, DDMAT and AIBN with the molar ration of 100 : 1 : 0.2 in the act of monomer, CTA and initiator respectively were used for the preparation of PNIPAM in dioxane with the phase transition ≃32 °C. PNIPAM (DP_th_ = 100) was identified using multiple techniques *i.e.* FT-IR, NMR, TGA and SEM.

The PNIPAM's IR spectrum represented the absorption peak of amide group (CO–NHR), NH bending, carbonyl group (C=O) and C–N stretching at 3311, 1542, 1654 and 1459 cm^−1^ respectively (Fig. A1, ESI†). The existence of the trithiocarbonate functionality at the PNIPAM's end group was demonstrated using ^1^H-NMR spectroscopy through the depiction of a peak at 3.7 ppm (CH_3_–(CH_2_)_10_–**CH**_**2**_–S–), 0.86 ppm (**CH**_**3**_–(CH_2_)_10_–CH_2_–S–) and 11.3 ppm (–C(CH_3_)_2_–**CO**_**2**_**H**) (Fig. A2, ESI†). In addition, average degree of polymerization (DP_*n*, NMR_) derived by comparing signal intensity at 3.7 ppm of the dodecylthiocarbonate group with integration at 4 ppm (–NH–C**H**(CH_3_)_2_) or 7.1 ppm (–N**H**–CH(CH_3_)_2_) of the NIPAM repeating group (Fig. A2, ESI†). The results expressed proper accord between experimental DP and theoretical ones.

**Fig. 1:**
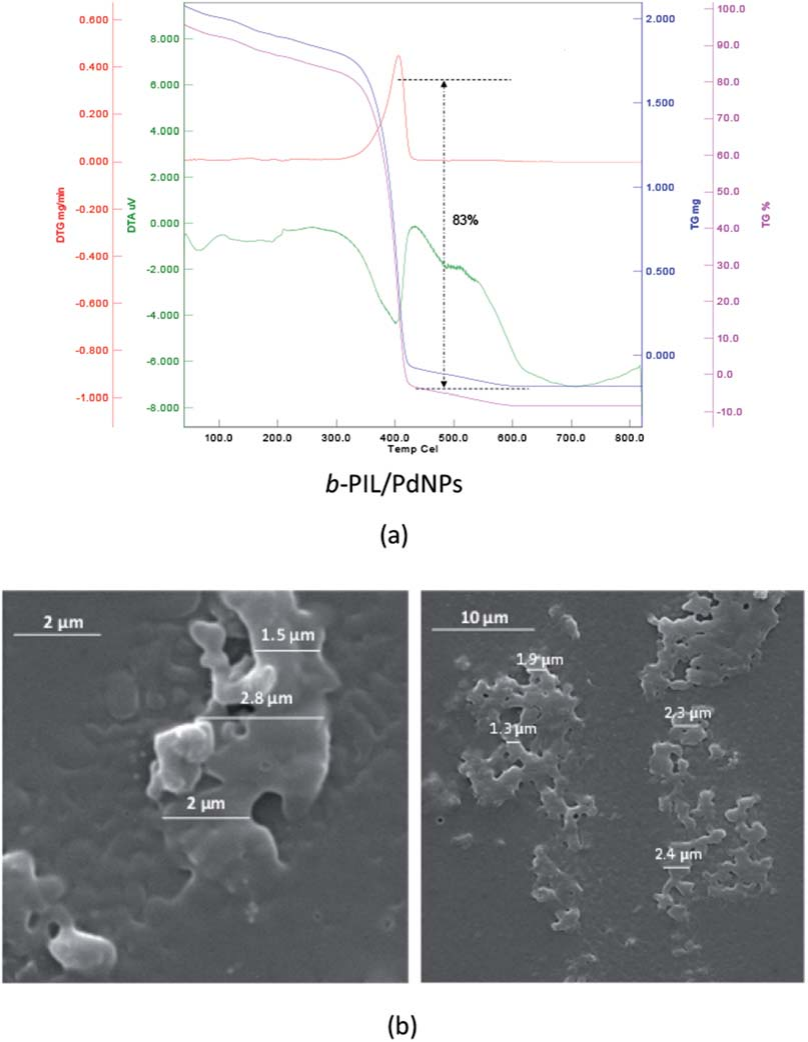
(a) TG, DTG and DTA data and (b) SEM images of PNIPAM (I).

Thermo gravimetric (TG) data of the PNIPAM (I) (heat rate of 10 °C min^−1^ from 25 to 800 °C in N_2_/O_2_ atmosphere) is depicted in Fig. 1a. The thermal deterioration of PNIPAM was took place in one main pyrolysis step. The beginning minor amount of weight loss was ascribed to the elimination of adsorbed small molecules and water. The PNIPAM manifested good thermal stability up to 300 °C. The weight markedly reduced around 350 °C up to 400 °C and organic components were decomposed completely over 410 °C. In addition, the scanning electron microscopy (SEM) image demonstrated the macroscopic structure, morphology and size of PNIPAM (Fig. 1b). The PNIPAM's SEM photograph emerged almost amorphous particles with nearly 2 μm dimension.

**Scheme 2:**
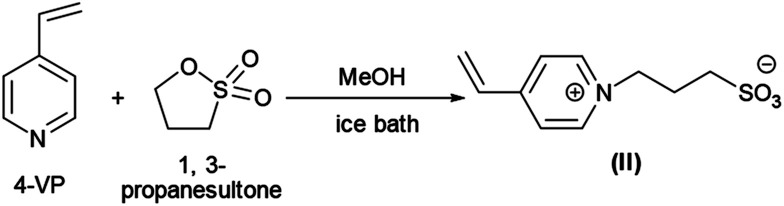
Illustrative preparation of ionic liquid monomer (II).

PNIPAM as macro-CTA was used to synthesize PNIPAM-*b*-PIL (III) through the reaction with ionic liquid monomer (II) (Scheme 1). 4-Vinyl pyridinium propane sulfonate (II) was produced formerly throughout the reaction of 4-vinyl pyridine and 1,3-propanesultone (Scheme 2). FT-IR spectrum of IL monomer (II) demonstrated the required characteristic peaks *i.e.* stretching vibration absorption of C=N (1519 cm^−1^) and C=C bond of the pyridine rings (1472 cm^−1^ and 1644 cm^−1^) and S=O asymmetric and symmetric stretching absorption of the –SO_3_^−^ group (1184 cm^−1^ and 1036 cm^−1^) (Fig. A3, ESI†). In addition, ^1^H-NMR spectrum of the IL monomer (II) is revealed the signals of the aromatic hydrogens of the pyridine ring at 7.5–9 ppm (2H appeared at 7.7 ppm and the other 2H ones at 8.6 ppm), vinyl hydrogens at 5.8, 6.3 and 6.8 ppm and aliphatic hydrogens at 1.7 ppm appropriately (Fig. A4, ESI†).

**Fig. 2:**
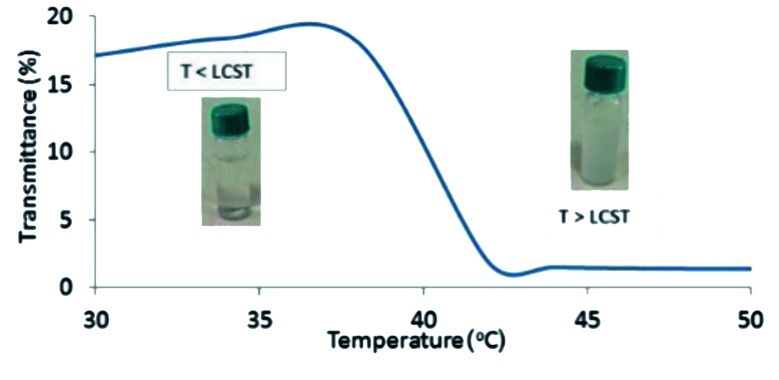
UV-Vis spectrum of PNIPAM-*b*-PIL (III) respecting temperature changes based on transmittance percentage.

In order to disclose the phase transition of PNIPAM-*b*-PIL (III), the transmittance of the block copolymer solution in deionized water was measured as a function of temperature using a UV-Vis spectrophotometer at 330 nm (Fig. 2). Cloud point of PNIPAM-*b*-PIL (III) occurred at around 40 °C that showed a shift compare to the homopolymer of PNIPAM (≃32 °C). It is expected that PIL as hydrophilic *co*-monomer increases the PNIPAM's phase transition. Above the lower critical solution temperature (LCST), the hydrophobic interactions become dominant and the cloudiness of the solution is clearly visible, owing to the collapse of the polymer structure. In addition, conversion of PNIPAM (I) to PNIPAM-*b*-PIL (III) is accompanied by the change in zeta potential from −93.4 to + 21.4.

FT-IR spectrum of PNIPAM-*b*-PIL (III) expressed absorption of amide group (CO–NHR) at 3322 cm^−1^, (NH) bending at 1541 cm^−1^, carbonyl functionality (C=O) at 1643 cm^−1^, C=C bond of aromatic ring at 1467 cm^−1^ and S=O asymmetric and symmetric absorption of the –SO_3_^−^ functionality at 1181 cm^−1^ and 1036 cm^−1^ respectively (Fig. A5, ESI†). Furthermore, ^1^H-NMR spectrum of PNIPAM-*b*-PIL (III) indicated the signal of the hydrogens of the pyridine ring at 7–7.5 ppm and the signal of hydrogen of isopropyl of PNIPAM at 3.7 ppm accordingly (Fig. A6, ESI†). The ratio integration for peaks of pyridine ring of PIL to isopropyl of PNIPAM is 1.1 : 1; therefore, we conclude that the DP of the PNIPAM block (DP = 100) is nearly quadruple than the DP of the PIL block (DP = 27). This may be due to the lower activity of the IL monomer (II) compare to NIPAM in this RAFT polymerization condition.

**Fig. 3:**
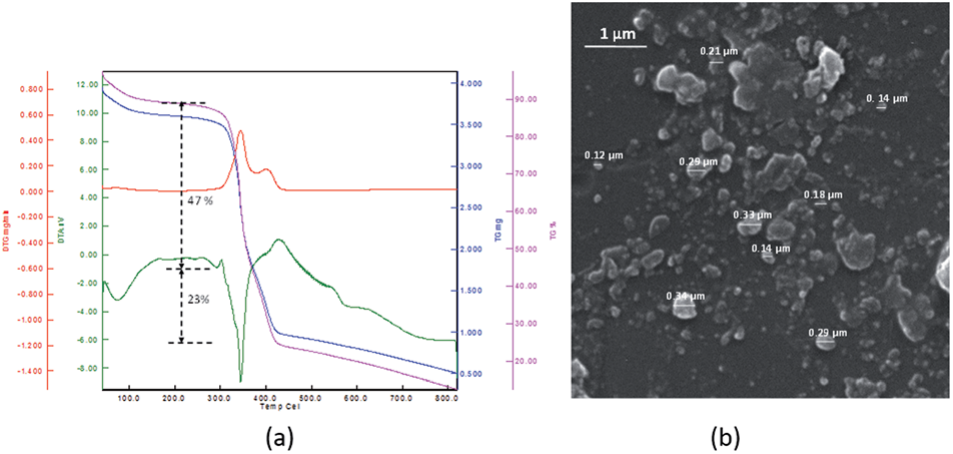
(a) TG, DTG and DTA data and (b) SEM image of PNIPAM-*b*-PIL (III).

TGA diagram of PNIPAM-*b*-PIL (III) under the same condition as PNIPAM is represented in Fig. 3a. The little amount of weight loss around 100 °C was corresponded completely to the removal of adsorbed H_2_O. The TGA graph displayed two pyrolysis stages due to the presence of two different blocks. The PNIPAM block decomposed nearly at 350 °C and PIL block at around 400 °C. The integration of PIL block is less than of PNIPAM block that matched to ^1^H-NMR data. Furthermore, the SEM image of PNIPAM-*b*-PIL (III) is outlined in Fig. 3b and illustrated the particles with quasi-spherical morphology and size range of 0.12–0.33 μm.

**Fig. 4:**
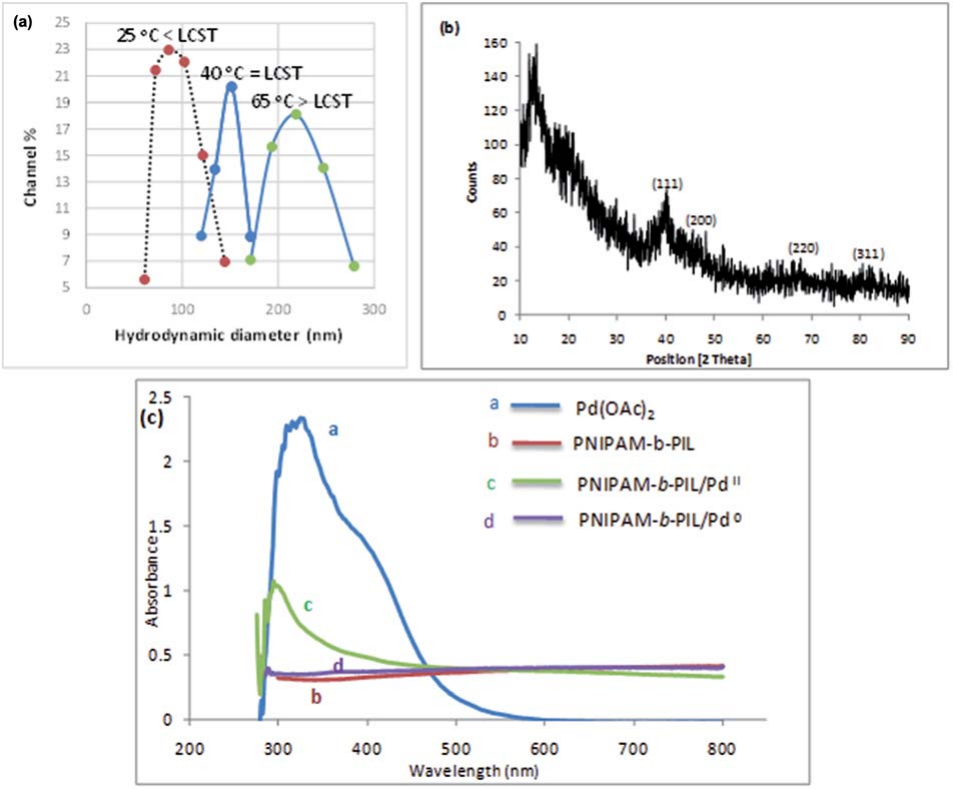
(a) Hydrodynamic size distribution plot of Pd catalyst (IV) before, after and at its LCST, (b) XRD spectrum of Pd catalyst (IV) and (c) UV-Vis illustration of Pd(OAc)_2_, PNIPAM-*b*-PIL (III), PNIPAM-*b*-PIL/Pd^II^ and PNIPAM-*b*-PIL/Pd^0^ catalyst (IV).

Finally, PNIPAM-*b*-PIL/Pd catalyst (IV) was generated by treating PNIPAM-*b*-PIL (III) with palladium acetate salt and subsequent reducing of Pd(ii) to Pd(0) with NaBH_4_ (Scheme 1). ICP analysis disclosed 0.196 mmol g^−1^ Pd loading. DLS experiment indicated approximate hydrodynamic diameter of this catalyst (IV) before, after and at its LCST (Fig. 4a). At ambient temperature of 25 °C (before its LCST) average size of 100 nm is observed, while at its LCST (40 °C) and after its LCST (65 °C) aggregates of approximately 140 and 220 nm were found respectively. Moreover, wide angle powder X-ray diffraction (XRD) motif of Pd catalyst is displayed in Fig. 4b. Crystallographic planes of Pd^0^ ((111), (200), (220) and (311)) was emerged at 2*θ* = 40.00, 46.49, 67.90 and 81.85 respectively and the mean size of the PdNPs (roughly calculated from the Scherrer equation) was found to be nearly 27 nm. In addition, the UV-Vis spectra of Pd(OAc)_2_, PNIPAM-*b*-PIL (III), PNIPAM-*b*-PIL/Pd^II^ and PNIPAM-*b*-PIL/Pd^0^ catalyst (IV) are represented in Fig. 4c. The spectrum of Pd(OAc)_2_ exhibited a peak at 328 nm while no peak was observed for PNIPAM-*b*-PIL (III). Binding the Pd to the block copolymer result in a peak appearance at 294 nm which is attributed to Pd(ii). After reduction step, the peak is disappeared which confirmed the Pd(ii) to Pd(0) conversion.

**Fig. 5:**
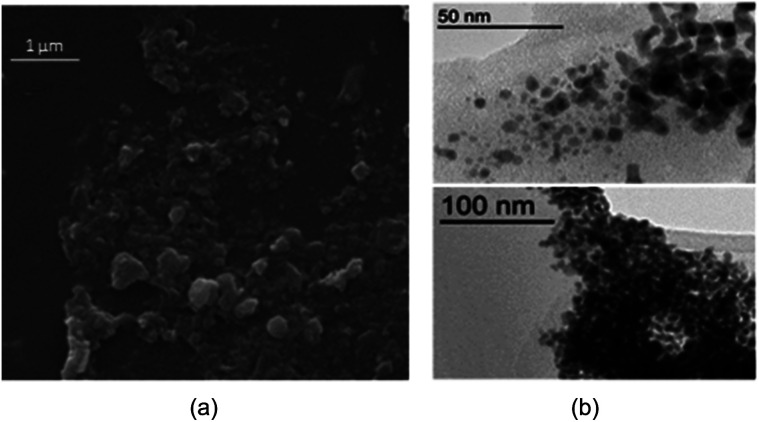
(a) SEM image and (b) TEM images of Pd catalyst (IV).

Fig. 5a is depicted the SEM photograph of PNIPAM-*b*-PIL/Pd catalyst (IV). The morphology (supposed spherical) and size (0.15 to 0.2 μm) of the catalyst do not revealed significant change compare to the block copolymer. Additionally, EDX spectrum of catalyst (IV) demonstrated 1.98% Pd loaded on the block copolymer. TEM picture of the Pd catalyst (IV) is rendered in Fig. 5b. The catalyst has a core (PdNPs)-corona (block copolymer) structure with the PdNPs' average size of 20 nm. No agglomeration of PdNPs occurred due to the electrostatic stabilization of PIL segments and steric stabilization of polymer chains.^54^

### 3.2. Mizoroki–Heck and Suzuki–Miyaura coupling reactions using PNIPAM-*b*-PIL/Pd catalyst

#### 3.2.1. Optimization of reaction condition

The impact of different variables in a Heck coupling reaction *i.e.* solvent, base and amount of polymeric Pd catalyst was studied in an exemplar reaction between phenyl iodide and *n*-butyl acrylate. The influence of dissimilar percentage of Pd catalyst was evaluated applying 0.01–1.5 mol% palladium amount. To explore the base potency, series of Heck reactions were fulfilled using K_2_CO_3_, Na_2_CO_3_, Et_3_N, Cs_2_CO_3_, NaOH and KOH as a base. Finally, efficacy of solvent on this reaction was probed as different media. The outcomes are collected in Table 1.

**Table 1:** Impact of base, solvent and Pd catalyst percentage for coupling of phenyl iodide and *n*-butyl acrylate^a^

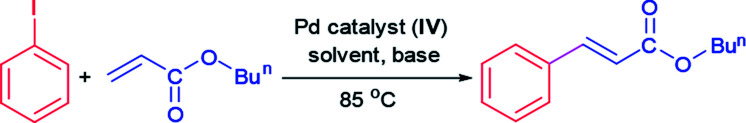					
No	Solvent	Base	Pd catalyst (mol%)	Time (min)	Conversion^b^ (%)
1	DMF	K_2_CO_3_	1.5	10	98
2	DMF	K_2_CO_3_	1	20	97
3	DMF	K_2_CO_3_	0.5	20	97
4	DMF	K_2_CO_3_	0.25	25	97
5	DMF	K_2_CO_3_	0.1	35	95
6	DMF	K_2_CO_3_	0.05	45	95
7	DMF	K_2_CO_3_	0.01	45	90
8	DMF	Na_2_CO_3_	1	15	95
9	DMF	NaOH	1	15	96
10	DMF	KOH	1	10	98
11	DMF	Cs_2_CO_3_	1	25	65
12	DMF	Et_3_N	1	15	97
13	DMF	Base free	1	180	—
14	H_2_O	Et_3_N	1	120	95
15	Dioxane	Et_3_N	1	50	65
16	Ethanol	Et_3_N	1	40	90
17	CH_2_Cl_2_	Et_3_N	1	40	10
18	H_2_O/ethanol	Et_3_N	1	100	50
19	H_2_O/DMF	Et_3_N	1	20	97
20^c^	H_2_O	Et_3_N	1	20	98
21^d^	H_2_O	Et_3_N	1	15	95
22	Solvent free	Et_3_N	1	15	94

a Optimization reactions are accomplished using 1.0 eq. iodobenzene, 1.2 eq. *n*-butyl acrylate, 2.0 eq. base and 2 mL of solvent at 85 °C.

b According to iodobenzene conversion.

c 0.5 mL H_2_O.

d TBAB (tetra-*n*-butylammonium bromide) as an additive.

The influence of catalyst quantity on the coupling of phenyl iodide and *n-*butyl acrylate exhibited that the catalyst loading can be minimized up to 0.01 mol% of Pd catalyst while the reaction time extended to 45 min (Table 1, entry 1–7). Performing the model reaction in different bases (using DMF as solvent and 1 mol% Pd catalyst) revealed Et_3_N as the best possibility (Table 1, entry 2 and entries 8–12). No coupling product was formed without base (Table 1, entry 13). Among various solvents and mixture of solvents, DMF was selected as the most appropriate one (Table 1, entry 12 and entries 14–19). Applying water as a green reaction medium is extremely important, in spite of the fact that, performing the reaction in H_2_O was generated the product after 120 min (Table 1, entry 14). Moreover, utilizing fewer amount of water as solvent (0.5 mL) or employing TBAB as additive yielded the superior result (Table 1, entries 20, 21). In addition, the reaction was carried out under solvent free condition promisingly (Table 1, entry 22).

#### 3.2.2. Mizoroki–Heck coupling reaction

The scope of Heck reaction using PNIPAM-*b*-PIL/Pd catalyst (IV) was shown using miscellaneous haloarenes include iodobenzene, bromobenzene, aryl iodides and bromides with electron-rich substituent (4-iodophenol, 1-iodo-4-methylbenzene, 1-iodo-4-methoxybenzene and 4-bromophenol), aryl iodides and bromides with electron-poor substituent (1-iodo-4-nitrobenzene, 1-bromo-4-iodobenzene, 1-chloro-4-iodobenzene, 1-bromo-4-nitrobenzene), substrates with *ortho* substitution (1-iodo-2-methylbenzene and 2-bromophenol) and *n*-butyl acrylate or styrene as olefinic compounds. The results are gathered in Table 2. Furthermore, 2-iodothiophene as a heterocyclic iodoarene was coupled with *n*-butyl acrylate and styrene and produced the products with 70% yield after 240 and 180 min respectively.

**Table 2:** Heck coupling of haloarenes with olefins using Pd catalyst (IV) in DMF^a^

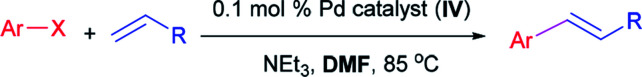						
No	Ar/X	R	Time (min)	TON^b^	TOF (min^−1^)^c^	Isolated yield^d^ (%)
1	Ph/I	CO_2_Bu^*n*^	30	960	32	96
2	4-OHPh/I	CO_2_Bu^*n*^	160	940	5.9	94
3	4-CH_3_Ph/I	CO_2_Bu^*n*^	15	930	62	93
4	2-CH_3_Ph/I	CO_2_Bu^*n*^	20	970	48.5	97
5	4-NO_2_Ph/I	CO_2_Bu^*n*^	60	940	15.7	94
6	4-BrPh/I	CO_2_Bu^*n*^	30	970	32.3	97
7	4-ClPh/I	CO_2_Bu^*n*^	30	960	32	96
8	4-OCH_3_Ph/I	CO_2_Bu^*n*^	12	900	75	90
9	4-OHPh/Br	CO_2_Bu^*n*^	420	950	2.3	95
10	4-NO_2_Ph/Br	CO_2_Bu^*n*^	540	850	1.6	85
11	2-OHPh/Br	CO_2_Bu^*n*^	420	800	1.9	80
12	Ph/Br	CO_2_Bu^*n*^	360	800	2.2	80
13	Ph/I	Ph	35	950	27.1	95
14	4-OHPh/I	Ph	150	800	5.3	80
15	4-CH_3_Ph/I	Ph	120	910	7.6	91
16	2-CH_3_Ph/I	Ph	300	800	2.7	80
17	4-NO_2_Ph/I	Ph	120	820	6.8	82
18	4-BrPh/I	Ph	120	960	8	96
19	4-ClPh/I	Ph	120	920	7.7	92
20	4-OCH_3_Ph/I	Ph	120	800	6.7	80

a All coupling reactions were accomplished with a molar ratios of ArX : olefin : NEt_3_ : Pd catalyst (IV) = 1.0 : 1.2 : 2.0 : 0.001 using 2 mL of DMF at 85 °C.

b TON = mmol of product/mmol of Pd in the catalyst.

c TOF = TON/time (min).

d Product identification was performed *via* their FT-IR and NMR spectra.

Since numerous beneficial features of water as an alternative to organic solvents, we resolved to examine the Heck coupling reaction in water as green reaction media. Diverse iodoarenes include substrates with electron-withdrawing and electron donating functionalities are coupled with *n*-butyl acrylate or styrene utilizing Pd catalyst (IV). The results are organized in Table 3. Furthermore, 2-iodothiophene is reacted with *n*-butyl acrylate successfully and provided the corresponding product in 96% yield after 240 min.

**Table 3:** Heck coupling of iodoarenes with olefins using Pd catalyst (IV) in H_2_O^a^

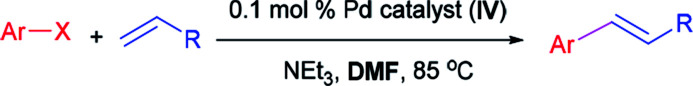						
No	Ar	R	Time (min)	TON	TOF (min^−1^)	Isolated yield^b^ (%)
1	Ph	CO_2_Bu^*n*^	35	940	26.8	94
2	2-CH_3_Ph	CO_2_Bu^*n*^	360	930	2.6	93
3	4-OCH_3_Ph	CO_2_Bu^*n*^	210	300	1.4	30
4	4-OHPh	CO_2_Bu^*n*^	240	800	3.3	80
5	4-CH_3_Ph	CO_2_Bu^*n*^	420	800	1.9	80
6	4-NO_2_Ph	CO_2_Bu^*n*^	150	950	6.3	95
7	4-BrPh	CO_2_Bu^*n*^	240	970	4	97
8	Ph	Ph	180	440	2.4	45
9	4-OCH_3_Ph	Ph	180	500	2.8	50
10	4-OHPh	Ph	120	500	4.2	50
11	4-NO_2_Ph	Ph	240	960	4	96
12	4-BrPh	Ph	30	200	6.7	20

a All coupling reactions were accomplished with a molar ratios of ArI : olefin : NEt_3_ : Pd catalyst (IV) = 1.0 : 1.2 : 2.0 : 0.001 using 0.5 mL of H_2_O at 85 °C.

b Product identification was performed *via* their FT-IR and NMR spectra.

**Scheme 3:**
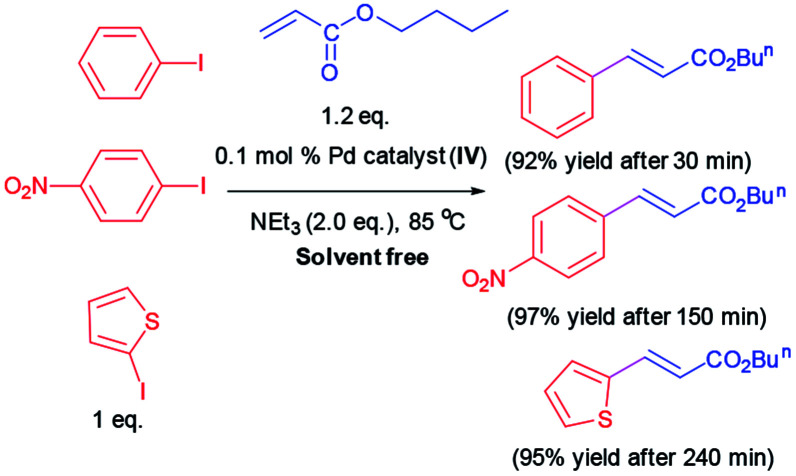
Heck reaction under solvent free condition.

Additionally, some Heck reactions are performed under solvent free condition in order to expand the capacity of our catalytic methodology (Scheme 3).

#### 3.2.3. Suzuki–Miyaura coupling reaction

The potency of the Pd catalyst (IV) was then explored in the Suzuki coupling reaction for the biaryls production. Various iodo and bromo-arenes were reacted with phenylboronic acid in H_2_O. The results of Suzuki coupling are tabulated in Table 4.

**Table 4:** Suzuki reaction of haloarenes with phenyl boronic acid using Pd catalyst (IV) a

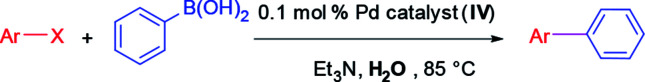						
No	Ar	X	Time (min)	TON	TOF (min^−1^)	Isolated yield^b^ (%)
1	Ph	I	20	950	47.5	95
2	4-OCH_3_Ph	I	40	960	24	96
3	4-CH_3_Ph	I	80	950	11.9	95
4	2-CH_3_Ph	I	90	960	10.7	96
5	4-ClPh	I	15	970	64.7	97
6	4-NO_2_Ph	I	40	940	23.5	94
7	4-CH_3_Ph	Br	120	920	7.7	92
8	2-CH_3_Ph	Br	180	900	5	90
9	4-OHPh	Br	180	700	3.9	70
10	2-OHPh	Br	720	700	1	70
11	4-NO_2_Ph	Br	180	920	5.1	92

a All coupling reactions were accomplished with a molar ratios of ArX : phenyl boronic acid : NEt_3_ : Pd catalyst (IV) = 1.0 : 1.2 : 2.0 : 0.001 using 0.5 mL of H_2_O at 85 °C.

b Products identification was performed *via* their FT-IR and NMR spectra.

### 3.3. The recyclability experiments of the catalyst

**Fig. 6:**
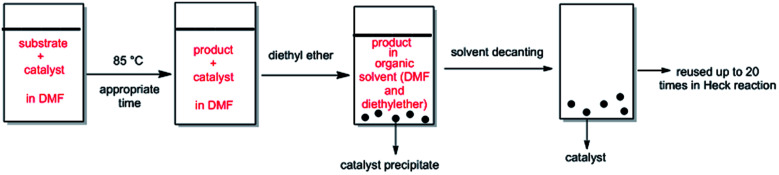
Schematic procedure of catalyst recycling in DMF.

**Fig. 7:**
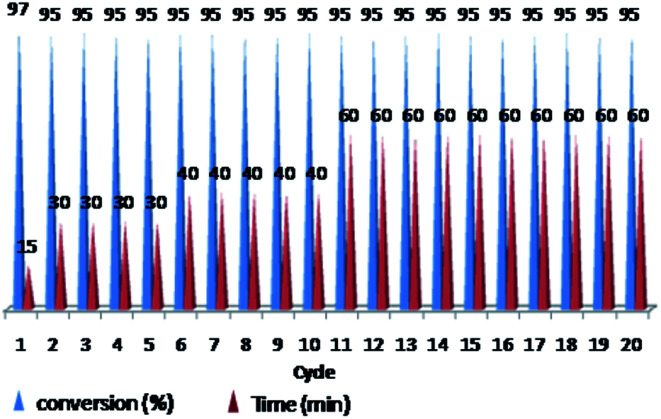
Reusability of PNIPAM-*b*-PIL/Pd catalyst (IV) in DMF (iodobenzene: *n*-butyl acrylate: Et_3_N: catalyst (IV) with a molar ratios of 1.0 : 1.2 : 2.0 : 0.01 at 85 °C).

The ability of precious metal catalyst's recycling with simple procedure and low leaching is very crucial from commercial and economical standpoint. To probe the reusability of the catalyst, the coupling Heck reaction of phenyl iodide with *n*-butyl acrylate in DMF was investigated for catalyst (IV). For this purpose, after fulfilment of the reaction, the suspension was chilled to room temperature and diethyl ether was added to the corresponding mixture. The product was remained in organic phase and the catalyst was precipitated (Fig. 6). After solvent decanting, the recovered catalyst was used in 20 subsequent reactions successfully without appreciable change in its efficiency (Fig. 7).

**Fig. 8:**
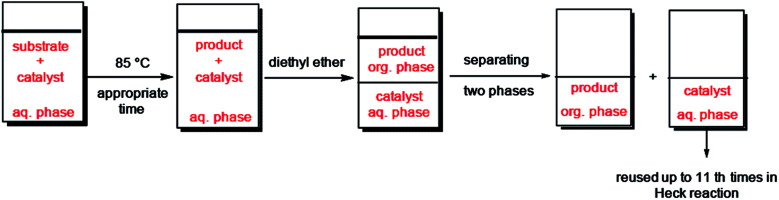
Schematic procedure of catalyst recycling in H_2_O.

**Fig. 9:**
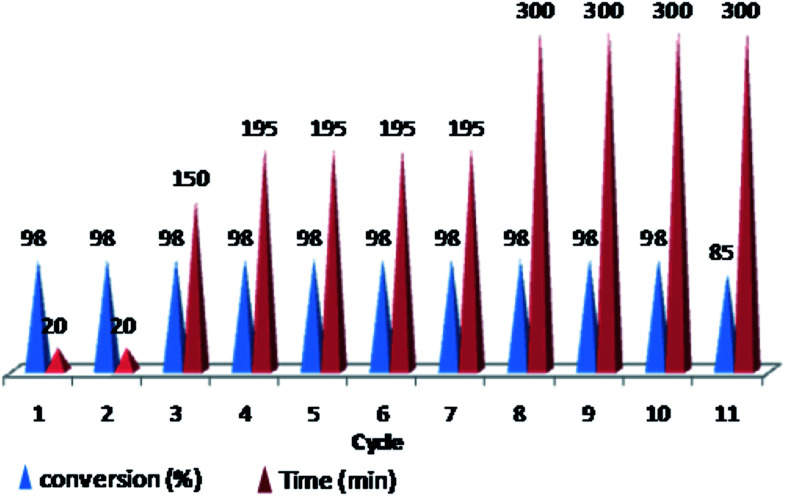
Reusability of PNIPAM-*b*-PIL/Pd catalyst (IV) in H_2_O (iodobenzene: *n*-butyl acrylate: Et_3_N: catalyst (IV) with a molar ratios of 1.0 : 1.2 : 2.0 : 0.01 at 85 °C).

Furthermore, we probed the reusability of the Pd catalyst (IV) in H_2_O. In this regard, after completion of a Heck reaction between phenyl iodide and *n*-butyl acrylate, diethyl ether was added to the suspension. The product extracted into organic phase and the catalyst remained in water. Aqueous and organic phases are separated and the recycled catalyst in water reused eleventh times in successive reactions without appreciable loss of its catalytic activity in these repeating cycles (Fig. 8 and 9).

**Fig. 10:**
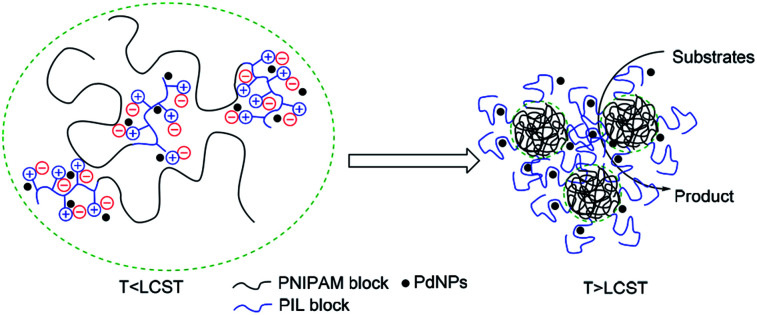
Schematic illustration of the Heck reaction within the PNIPAM-*b*-PIL/PdNPs in water.

Effective catalytic activity of PNIPAM-*b*-PIL/PdNPs in water at 85 °C *i.e.* the temperature above the LCST of PNIPAM is related to the diffusion of the reactants into collapsed PNIPAM's chain due to its micellization.^55,56^ Actually, PNIPAM-*b*-PIL/PdNPs as a nanoreactor offer an extremely high concentration of substrates around the PdNPs as shown in Fig. 10.

Investigation the catalyst recycling for the Suzuki coupling of iodobenzene and phenyl boronic acid undergoing optimized reaction condition also revealed successful reusing of the catalyst at the minimum of ten times in successive reactions without remarkable depletion of its activity *i.e.* no decrease of product yields and time increasing from 20 min to 40 min.

In addition, to explore the amount of palladium leaching from the support, the Pd content (mmol g^−1^) of the catalyst was determined after 10 repeating cycles in Heck coupling reaction and compared with Pd content of fresh catalyst. It is reduced from 0.196 mmol g^−1^ to 0.176 mmol g^−1^ and therefore, indicated low Pd leaching from the support.

## 4. Conclusion

In the present research, the synthesis of thermo-responsive PNIPAM-*b*-PIL based on modified vinylpyridine-type ionic liquid *via* RAFT technique is reported. RAFT polymerization imparted a facile process to prepare block copolymer with foreseeable molecular weights. PNIPAM-*b*-PIL supported PdNPs is prepared *via* Pd(OAc)_2_ treatment. The catalyst was identified thoroughly by ICP, UV-Vis spectrophotometer, TGA, XRD, SEM and TEM analysis. The PNIPAM-*b*-PIL/PdNPs was employed in the Heck and Suzuki coupling for the production of various substituted alkenes and biaryls in a moderate reaction condition. The coupling reactions can be performed in organic, aqueous or even under solvent free condition. Effective catalytic activity of PNIPAM-*b*-PIL/PdNPs in water at the temperature above the LCST of PNIPAM is related to the diffusion of the reactants into collapsed PNIPAM's chain. The catalyst displayed extreme recyclability with simple procedure and low leaching.

## Conflicts of interest

There are no conflicts to declare.

## Supplementary Material

RA-008-C8RA01303A-s001
