# Supplementary material for: Thermo-responsive poly(N-isopropylacrylamide)-block-poly(ionic liquid) of pyridinium sulfonate immobilized Pd nanoparticles in C–C coupling reactions
Source: RSC Adv. 2018 Apr 18;8(26):14570–8. doi: 10.1039/c8ra01303a (PMC9079935; doi:10.1039/c8ra01303a)
Supplement: RA-008-C8RA01303A-s001 [file RA-008-C8RA01303A-s001.pdf]

Electronic Supplementary Material (ESI) for RSC Advances.
This journal is © The Royal Society of Chemistry 2018

Supplementary Information

Thermo-responsive poly(*N*-isopropylacrylamide)-*block*-poly (ionic liquid) of pyridinium sulfonate immobilized Pd nanoparticles in C-C coupling reactions

Soheila Ghasemi* and Zahra Amini Herandi

Department of Chemistry, College of Sciences, Shiraz University, Shiraz, Iran

Corresponding Author: Soheila Ghasemi, Tel.: *+98-713-6460724*; Fax: *+98-713-6460788*; E-mail address: *ghasemis@shirazu.ac.ir* ; Postal address: Department of Chemistry, College of Sciences, Shiraz University, Shiraz *7194684795*, Iran.

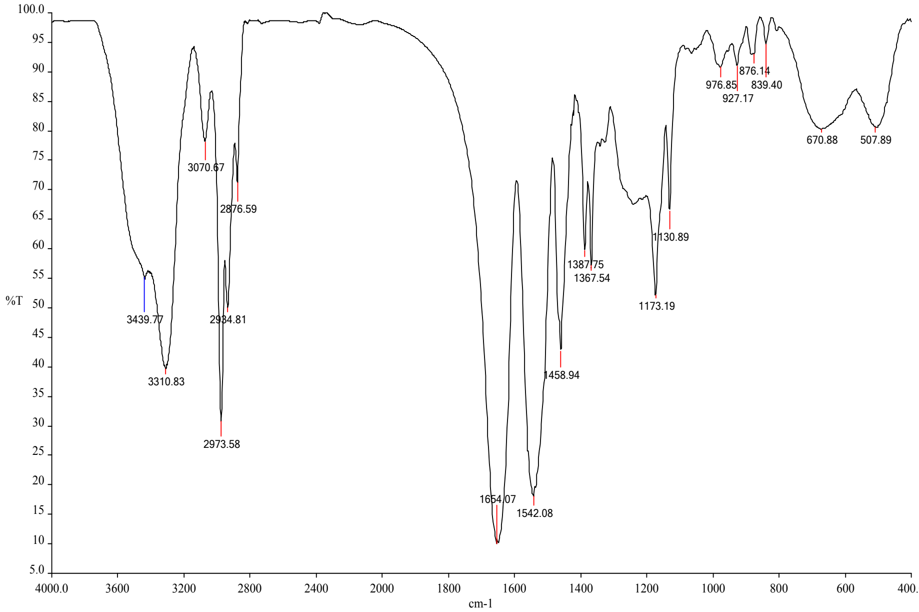

Figure A1 FT-IR spectrum of PNIPAM **(I)**

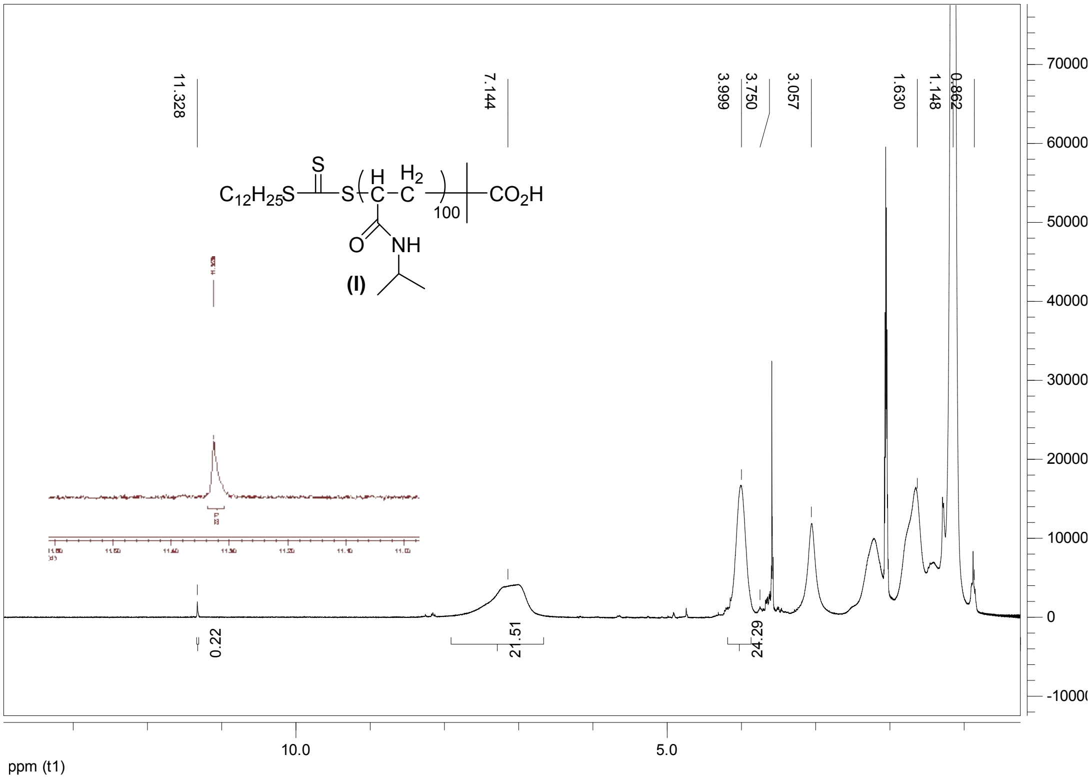

Figure A2 ^{1}H-NMR spectrum of PNIPAM (**I**) in aceton-d_6

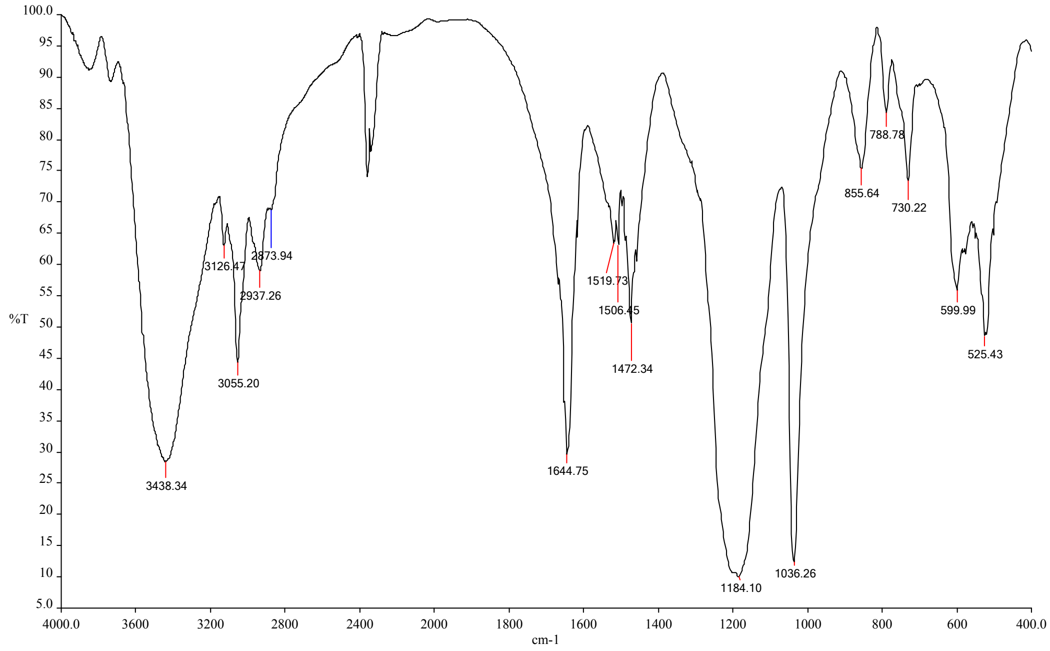

Figure A3 FT-IR spectrum of ionic liquid monomer (**II**)

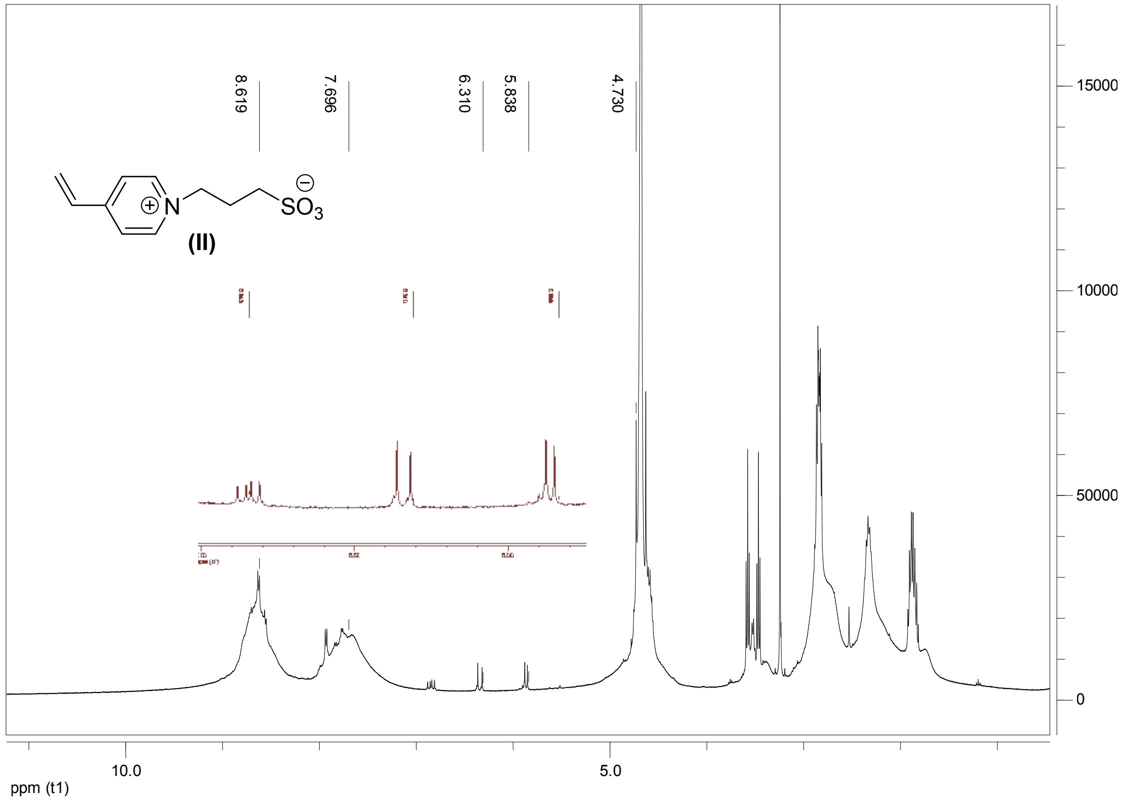

Figure A4^{1}H-NMR spectrum of ionic liquid monomer (**II**) in D_2O

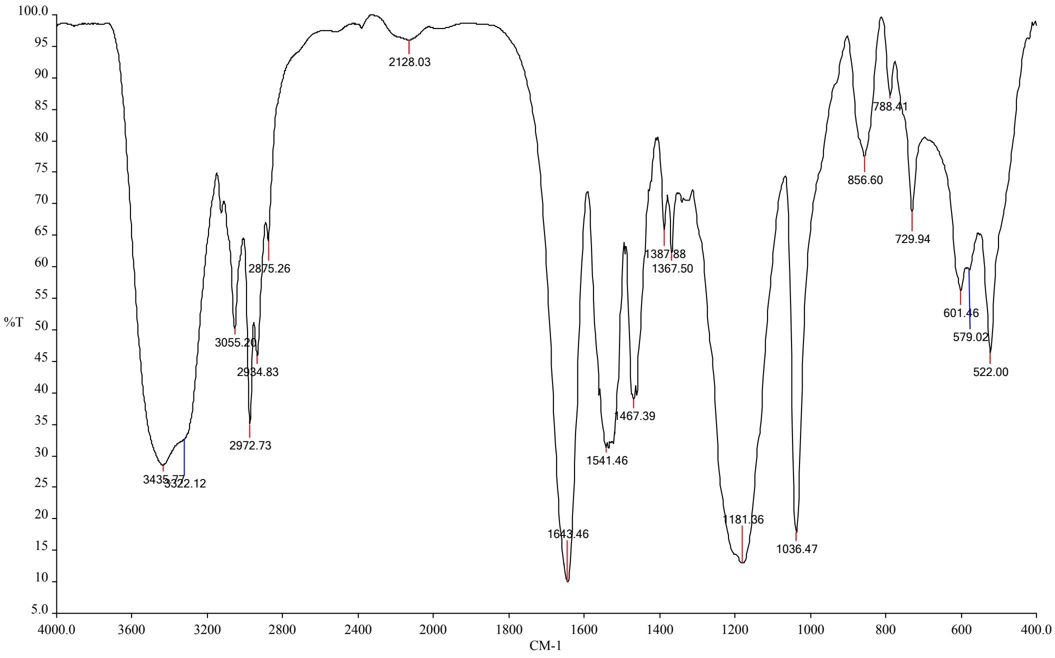

Figure A5 FT-IR spectrum of PNIPAM-*b*-PIL (**III**)

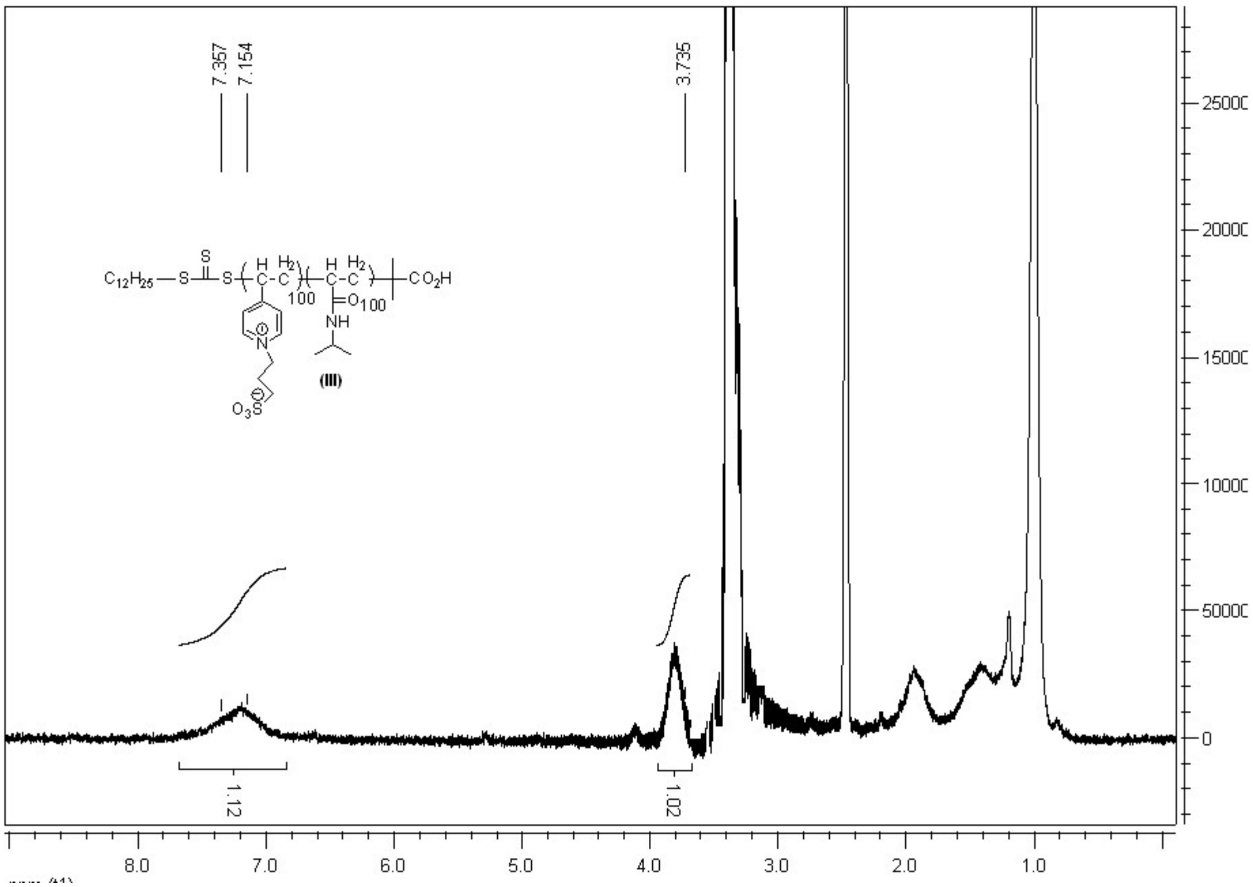

Figure A6 ^{1}H-NMR spectrum of PNIPAm-*b*-PIL (**III**) in D_2O
